# Supplementary material for: Gene Network Analysis of Alzheimer’s Disease Based on Network and Statistical Methods
Source: Entropy (Basel). 2021 Oct 19;23(10):1365. doi: 10.3390/e23101365 (PMC8535014; doi:10.3390/e23101365)
Supplement: Supplementary file 1 [file entropy-23-01365-s001.zip › entropy-1384317-supplementary.pdf]

Table S1 Herb compounds

Herb	Active compounds
KXS (kaixinsan)	Panax Ginseng C.A.Mey, Poria Cocos(Schw.)Wolf., Acoritataninowii Rhizoma
DGSYS (dangguishaoyaosan)	Poria Cocos(Schw.)Wolf., Angelicae Sinensis Radix, Paeoniae Radix Alba, Chuanxiong Rhizoma, Atractylodes Macrocephala Koidz., Alisma Orientale(Sam.)Juz.
YGS (yigansan)	Poria Cocos(Schw.)Wolf., Angelicae Sinensis Radix, Chuanxiong Rhizoma, Atractylodes Macrocephala Koidz., Radix Bupleuri, Uncariae Ramulus Cumuncis
YQTYT (yiqitongyutang)	Panax Ginseng C.A.Mey., Angelicae Sinensis Radix, Chuanxiong Rhizoma, Radix Salviae, Codonopsis Radix, Radix Paeoniae Rubra, Hedysarum Multijugum Maxim.

Table S2 Information of herb compound target genes

Compound	Target genes
Panax GinsengC.A.Mey.	ACHE, ACP3, ADCYAP1, ADH1A, ADH1B, ADH1C, ADRA1A, ADRA1B, ADRA1D, ADRA2A, ADRA2B, ADRA2C, ADRB1, ADRB2, AHR, AHSA1, AKR1C3, AKT1, ALOX5, AR, BAX, BCHE, BCL2, CACNA1S, CAMKK2, CASP1, CASP3, CASP8, CASP9, CDC2, CHRM1, CHRM2, CHRM3, CHRM4, CHRM5, CHRNA2, CHRNA7, CTRB1, CTSD, CYP1A1, CYP1A2, CYP1B1, CYP3A4, DIO1, DPP4, DRD5, F10, F2, F7, GABRA1, GABRA2, GABRA3, GABRA5, GABRA6, GLB1, GLRA1, GRIA2, GSK3B, GSTM1, GSTM2, GSTP1, HAS2, HMOX1, HSP90AA1, HTR2A, HTR3A, ICAM1, IFNG, IGHG1, IGHG2, IKBKB, IL1B, INSR, JUN, KCNH2, KCNMA1, KDR, LCT, LTA4H, MAN1A2, MAN2A1, MAOA, MAOB, MAP1B, MAP2, MAP2K4, MAPK14, MAPK8, MMP1, NCOA1, NCOA2, NFKBIA, NOS1, NOS2, NOS3, NR1I2, NR1I3, NR3C1, NR3C2, OPRD1, OPRM1, PDE3A, PGR, PIK3CG, PKIA, PLAU, PON1, PPARG, PPP3CA, PRKACA, PRKCA, PRSS1, PRSS3, PSMD3, PSMG1, PTGS1, PTGS2, PTPN1, PYGM, RELA, RHO, RXRA, RXRG, SCN5A, SELE, SLC2A4, SLC6A2, SLC6A3, SLC6A4, SLPI, STAT1, TGFB1, TNF, TOP-2, VCAM1, XDH
Acoritataninowii Rhizoma	ACHE, ADH1A, ADH1B, ADH1C, ADRA1A, ADRA1B, ADRA1D, ADRA2A, ADRA2B, ADRA2C, ADRB1, ADRB2, AHR, AHSA1, AKR1C3, AKT1, ALOX5, AR, BAX, BCL2, CA2, CAMKK2, CAMKK2 , CASP3, CCL16, CCNA2, CDK2, CHEK1, CHRM1, CHRM2, CHRM3, CHRNA2, CHRNA7, COBT, CTRB1, CYP1A1, CYP1A2, CYP1B1, CYP3A4, DIO1, DPP4, DRD1, DRD5, ESR1, ESR2, F10, F2,

	F7, FOS, GABRA1, GABRA2, GABRA3, GABRA3, GABRA5, GABRA6, GAD2, GRIA2, GSK3B, GSTM1, GSTM2, GSTP1, HAS2, HBA1, HMOX1, HSP90AA1, HSP90AB1, HTR2A, HTR2A , ICAM1, IKBKB, INSR, JUN, KCNH2, KCNMA1, KDR, LTA4H, LYZ, MAOA, MAOB, MAPK14, MAPK8, MMP1, NCOA2, NOR51B-261, NOS2, NOS3, NR1I2, NR1I3, NR3C2, PDE3A, PGR, PIK3CG, PIM1, PKIA, PLA2G2E, PLAU, PPARG, PPP3CA, PRKACA, PRSS1, PRSS3, PSMD3, PTGS1, PTGS2, PTPN1, PYGM, RELA, RXRA, SCN5A, SELE, SLC2A4, SLC6A2, SLC6A3, SLC6A4, SLPI, STAT1, TNF, TOP-2, TYR, TYRP1, VCAM1, XDH
Poria Cocos(Schw.) Wolf.	ADH1C, ADRA1B, BFRC, CHRM1, CHRM2, CHRM3, COBT, FABB, GABRA1, GABRA2, GABRA3, GABRA5, GABRA6, GRIA2, IGHG1, LYSOZ1, NCOA2, NOR51B_261, NR3C2, PDE3A, PGR, PLA2G1B, PTGS1, PTGS2, RXRA, SCN5A, SLC6A2
Angelice Sinensis Radix	ABAT, ACHE, ADH1A, ADH1B, ADH1C, ADRA1A, ADRA1B, ADRA1D, ADRA2A, ADRA2B, ADRA2C, ADRB1, ADRB2, AGXT, AGXT2, AKR1, ALDH5A1, APOB, ARG, ATP5F1B, BAX, BCHE, BCL2, CA2, CA2, CASP3, CASP8, CASP9, CAT, CHDH, CHRM1, CHRM2, CHRM3, CHRM4, CHRNA2, CHRNA7, CTH, CTRB1, CTSD, DHODH, DRD5, ESR1, F10, F13A1, F2, GABRA1, GABRA2, GABRA3, GABRA5, GABRA6, GARS1, GATM, GLB1, GNMT, GOT2, GPHN, GPT, GRIA2, GRIK2, HP, HTR2A, IGHG1, IGHG2, IL2, JUN, KCNH2, LPL, LTA4H, MAOB, MAP2, MAPK1, MARS1, ME2, MMP8, MMP9, MOCS1, MOGAT2, NCOA1, NCOA2, NFATC3, NFS1, NOS3, NR3C1, NR3C2, OPRM1, PDE3A, PDHX, PGR, PIK3CG, PIM1, PKIA, PLA2G2E, PLAU, PNP, PON1, PPARA, PPARGC1A, PPIF , PRKCA, PRSS3, PTGS1, PTGS2, PTPN1, RELA, RXRA, SCN10A, SCN5A, SLC36A1, SLC6A2, SLC6A3, SLC6A4, TGFB1, TKFC , TOP-2, TPI1, XDH
Chuanxiong Rhizoma	ABAT, ABI1, ACHE, ADH1A, ADH1C, ADRA1A, ADRA1B, ADRA1D, ADRA2A, ADRA2B, ADRA2C, ADRB1, ADRB2, AKR1, ALDH2, ANXA3, AR, ARG1, ASNS, BCHE, BDNF, CA2, CAT, CCK, CCNA2, CD36, CETP, CHAT, CHEK1, CHRM1, CHRM2, CHRM3, CHRM4, CHRNA2, CHRNA7, CITED1, CRP, CTRB1, DNPEP, DRD5, EDN1, ELANE, ENPEP, ERBB2, ESR1, ESR2, F10, F2, F3, F7, FABP1, FOS, GABRA1, GABRA2, GABRA3, GABRA5, GABRA6, GAD2, GAMT, GAP43, GARS1, GCG, GM2A, GNMT, GRIA2, GSK3B, GSTA1, HMGCR, HTR2A, HTR3A, IGHG1, IGHG2, INS, JUN, KCNH2, KDR, LPCAT2, LPL, LTA4H, MAN1A2, MAOB, MAPK1, MAPK10, MAPK14, MARS1, MMP8, MMP9, MPO, NCOA1, NCOA2, NOS2, NOS3, NR3C1, NR3C2, NTRK2, PAM, PDE3A, PDX1, PGR, PIK3CG, PKIA, PKM, PLA2G1B, PLA2G2E, PLAU, PLG, PNP, PON1, PPARA,

	PPARD, PPARG, PPIF , PRKACA, PRODH, PRSS3, PTGS1, PTGS2, PYY, RBP2, RXRA, SCD, SCN10A, SCN5A, SERPINE1, SLC2A2, SLC6A2, SLC6A3, SLC6A4, SLC7A7, SOAT1, SOD1, TEP1, TNF, TOP-2, TPI1, TRPV1, TYRP1, UCP2, UCP3, VEGFA
Paeoniae Radix Alba	ACHE, ADRA1A, ADRA1B, ADRB2, AHR, AHSA1, AKR1C3, AKT1, ALOX5, AR, BAX, BCL2, CAMKK2, CASP3, CASP8, CASP9, CAT, CD14, CDC2, CHRM1, CHRM2, CHRM3, CHRM4, CHRNA2, CHRNA7, CYP1A1, CYP1A2, CYP1B1, CYP3A4, DIO1, DPP4, DRD1, ESR1, F2, F7, GABRA1, GABRA2, GABRA3, GABRA5, GSTM1, GSTM2, GSTP1, HAS2, HMOX1, HSP90AB1, HTR2A, ICAM1, IKBKB, IL6, INSR, JUN, KCNH2, LBP, MAP2, MAPK8, MMP1, NCOA2, NOS2, NOS3, NR1I2, NR1I3, NR3C2, OPRM1, PDE3A, PGR, PIK3CG, PON1, PPARG, PPP3CA, PRKACA, PRKCA, PRSS1, PSMD3, PTGS1, PTGS2, RELA, RXRA, SCN5A, SELE, SLC2A4, SLC6A2, SLC6A4, SLPI, STAT1, TGFB1, TNF, TOP-2, VCAM1, XDH
Atractylodes Macrocephala Koidz.	ABAT, ACHE, ADH1A, ADH1B, ADH1C, ADRA1A, ADRA1B, ADRA2A, ADRA2B, ADRA2C, ADRB1, ADRB2, ADSS1, AGXT, AGXT2, AKR1, ALAS1, ALAS2, ALDH1A1, ALDH2, ALDH5A1, ALOX5, AMY2A, AR, ARG, ARG1, ARS, ATP2C1, BCAT1, BCHE, CA2, CAMKK2, CAT, CDC2, CDC25B, CHRM1, CHRM2, CHRM3, CHRM4, CHRNA2, CHRNA7, CKM, CPT2, CTH, CTRB1, CTSD, DAO, DHODH, DPP4, DRD1, DRD5, F10, F13A1, F2, F7, GABRA1, GABRA2, GABRA3, GABRA5, GABRA6, GALE, GARS1, GATM, GCAT, GFOD1, GLB1, GLDC, GLRA1, GLRA2, GLRA3, GLUD1, GLUD2, GLYAT, GLYATL1, GNMT, GOT1, GOT2, GPHN, GPI, GPT, GPT2, GRIA1, GRIA2, GRIK2, GRIN1, GRIN2A, GRIN2B, GRIN2C, GRIN3A, HSP90AA1, IGHG1, IGHG2, IL1B, IL6, KCNH2, KCNMA1, KYNU, LDHA, LDHB, LPCAT2, LTA4H, LTF, MAN1A2, MAOB, MARS1, MAT1A, MAT2A, ME1, ME2, ME3, MMP12, MMP8, MOCS1, MTR, NAGS, NCOA1, NCOA2, NFS1, NOS1, NOS3, NR3C1, NR3C2, OAT, OPLAH, OPRM1, OTC, P3H3, P4HA1, PC, PDE3A, PDHB, PFAS, PGF, PGR, PIK3CG, PIM1, PIPOX, PLA2G1B, PLA2G2E, PPARG, PPIA, PPIF , PRODH, PRSS3, PTER, PTGS1, PTGS2, PYGL, PYGM, REN, RRM1, RXRA, SAP18, SCN5A, SDHA, SHMT1, SHMT2, SLC25A10, SLC25A12, SLC25A13, SLC36A1, SLC6A2, SLC6A3, SLC6A4, SLC6A9, SRC, TKFC , TNF, TOP-2, TPI1, TXNRD1, UL30, VEGFA, XDH, YARS2
Alisma Orientale (Sam.) Juz.	ACHE, ADH1B, ADH1C, ADRA1B, CHRM1, CHRM2, CHRM3, CHRNA7, DPP4, F2, GABRA1, GABRA2, GABRA3, GABRA5, GABRA6, GRIA2, IGHG1, IGHG2, MAN2A1, NCOA2, NR3C1, NR3C2, PGR, PRSS3, PTGS1, PTGS2

Radix Bupleuri	ABCA2, ACACA, ACHE, ACP3, ADH1C, ADRA1A, ADRA1B, ADRA2A, ADRB1, ADRB2, AHR, AHSA1, AKR1C3, AKT1, ALOX5, AR, ARG, BAX, BCL2, BCL2L1, BIRC5, CASP3, CASP8, CASP9, CAV1, CCL2, CCNA2, CCNB1, CCND1, CD40LG, CDKN1A, CHEK1, CHEK2, CHRM1, CHRM2, CHRM3, CHRNA7, CHUK, CLDN4, COL1A1, COL1A3, CRP, CTNNBIP1, CTRB1, CTSD, CXCL10, CXCL11, CXCL2, CXCL8, CYP1A1, CYP1A2, CYP1B1, CYP3A4, DCAF5, DHODH, DIO1, DRD5, DUOX2, E2F1, E2F2, EGF, EGFR, EIF6, ELK1, ERBB2, ERBB3, ESR1, ESR2, F2, F3, F7, FOS, GABRA1, GABRA2, GABRA3, GABRA5, GARS1, GJA1, GRIA2, GRP78, GSK3B, GSTM1, GSTM2, GSTP1, HAS2, HIF1A, HK2, HMOX1, HSF1, HSPB1, HTR2A, ICAM1, IFNG, IGF2, IGFBP3, IGHG1, IGHG2, IKBKB, IL10, IL1A, IL1B, IL2, IL6, INSR, IRF1, JUN, KCNH2, LTA4H, MAOB, MAPK1, MAPK14, MAPK8, MARS1, MGAM, MMP1, MMP2, MMP3, MMP9, MPO, MYC, NCF1, NCOA1, NCOA2, NFE2L2, NFKBIA, NKX3-1, NOS2, NOS3, NPEPPS, NQO1, NR1I2, NR1I3, NR3C2, ODC1, OLR1, PCOLCE, PGR, PIK3CG, PIM1, PKM, PLAT, PLAU, PON1, POR, PPARA, PPARD, PPARD, PPARG, PPIF , PPP3CA, PRKCA, PRKCB, PRSS1, PSMD3, PTEN, PTER, PTGER3, PTGS1, PTGS2, PTPN1, PYGM, RAF1, RASA1, RASSF1, RB1, RELA, RUNX1T1, RUNX2, RXRA, SCN5A, SELE, SERPINE1, SLC2A4, SLC6A2, SLC6A3, SLPI, SOD1, SPP1, STAT1, STE, TGFB1, THBD, TNF, TNKS, TOP1, TOP-2, TOP2A, TP53, VCAM1, VEGFA, XDH
Uncarie Ramulus Cumuncis	AARS1, ABCA2, ACACA, ACHE, ACP3, ADH1A, ADH1C, ADRA1A, ADRA1B, ADRA1D, ADRA2A, ADRA2B, ADRA2C, ADRB1, ADRB2, AHR, AHSA1, AKR1, AKR1C3, AKT1, ALOX5, AR, ARG, BACE1, BAX, BCL2, BCL2L1, BIRC5, CA2, CASP3, CASP8, CASP9, CAV1, CCL2, CCNB1, CCND1, CD40LG, CDKN1A, CHEK1, CHEK2, CHRM1, CHRM2, CHRM3, CHRM4, CHRM5, CHRNA2, CHRNA7, CHUK, CLDN4, COL1A1, COL1A3, CRP, CTNNBIP1, CTSD, CXCL10, CXCL11, CXCL2, CXCL8, CYP1A1, CYP1A2, CYP1B1, CYP3A4, DAO, DCAF5, DHODH, DIO1, DRD2, DRD3, DRD5, DUOX2, E2F1, E2F2, EGF, EGFR, EIF6, ELK1, ERBB2, ERBB3, ESR1, ESR2, F10, F2, F3, F7, FOS, GABRA1, GABRA2, GABRA3, GABRA5, GABRA6, GARS1, GJA1, GRIA2, GRP78, GSK3B, GSTM1, GSTM2, GSTP1, HAS2, HIF1A, HK2, HMOX1, HSF1, HSPB1, HTR2A, HTR2C, HTR3A, HTR7, ICAM1, IFNG, IGF2, IGFBP3, IGHG1, IGHG2, IKBKB, IL10, IL1A, IL1B, IL2, IL6, INSR, IRF1, ISYNA1, JUN, KCNH2, KDR, LPCAT2 , LPL, MAOB, MAP2, MAPK1, MAPK8, MARS1, MDK, MET, MGAM, MMP1, MMP2, MMP3, MMP9, MPO, MYC, NCF1, NCOA1, NCOA2, NFE2L2, NFKBIA, NGF, NKX3-1, NOS2, NOS3, NPEPPS, NQO1, NR1I2, NR1I3, NR3C1, NR3C2, ODC1, OPRD1, OPRK1,

	OPRM1, PCOLCE, PDE10A, PDE3A, PGR, PIK3CG, PIM1, PKM, PLA2G2E, PLAT, PLAU, PON1, POR, PPARA, PPARD, PPARG, PPIF , PPP3CA, PRKCA, PRKCB, PRSS1, PRSS3, PSMD3, PTEN, PTER, PTGER3, PTGS1, PTGS2, PTPN1, RAF1, RASA1, RASSF1, RB1, RELA, RUNX1T1, RUNX2, RXRA, RXRB, SCN5A, SELE, SERPINE1, SLC2A4, SLC6A2, SLC6A3, SLC6A4, SLPI, SOD1, SPP1, STAT1, STE, TGFB1, THBD, TNF, TNKS, TOP1, TOP-2, TOP2A, TP53, VCAM1, VEGFA, XDH
Radix Salviae	AARS1, ACHE, ADCY2, ADRA1A, ADRA1B, ADRA1D, ADRA2A, ADRA2B, ADRA2C, ADRB2, AHSA1, AKT1, APP, AR, ARG, BCL2, BCL2L1, BIRC5, CALCR, CASP3, CASP7, CASP9, CCNA2, CCNB1, CCND1, CD40LG, CDKN1A, CHEK1, CHRM1, CHRM2, CHRM3, CHRM4, CHRM5, CHRNA2, CHRNA7, CYP1A1, CYP1A2, CYP3A4, DHODH, DRD2, DRD5, ECE1, EDN1, EDNRA, EGFR, ERBB2, ESR1, ESR2, F10, F2, F7, FASN, FOS, GABRA1, GABRA2, GABRA3, GABRA5, GABRA6, GABRE, GABRG3, GARS1, GSK3B, GSTP1, HMOX1, HTR1A, HTR1B, HTR2A, HTR2C, HTR3A, ICAM1, IFNG, IGHG1, IGHG2, IL10, IL2, IL4, IL6, INSR, ITGB3, JUN, KCNH2, LPCAT2 , MAPK1, MAPK14, MCL1, MDM2, MET, MMP1, MMP2, MMP9, MYC, NCOA1, NCOA2, NFKBIA, NOS2, NPM1, NR1I2, NR3C1, NR3C2, NUF2, OPRD1, OPRM1, PCNA, PDE3A, PGR, PIK3CG, PIM1, PKM, PPARG, PPIF , PRSS1, PTER, PTGES, PTGS1, PTGS2, PTPN1, RB1, RELA, RXRA, SCN5A, SLC2A4, SLC6A2, SLC6A3, SLC6A4, STAT3, TNF, TNKS, TOP1, TOP-2, TOP2A, TP53, TYR, VEGFA, XDH
Codonopsis Radix	AARS1, ACHE, ADCY2, ADH1C, ADRA1A, ADRA1B, ADRA1D, ADRA2A, ADRB1, ADRB2, AKT1, AR, ARG, BCL2L1, BIRC5, CASP3, CASP7, CASP9, CCNA2, CCNB1, CCND1, CD40LG, CDKN1A, CHEK1, CHRM1, CHRM2, CHRM3, CHRM5, CHRNA7, CTRB1, DHODH, DRD5, EGFR, ERBB2, ESR1, ESR2, F10, GABRA1, GABRA2, GABRA5, GSK3B, GSTP1, HMOX1, HTR2A, ICAM1, IFNG, IGHG1, IGHG2, IL10, IL2, IL4, IL6, INSR, JUN, LTA4H, MAOB, MAPK1, MAPK14, MARS1, MCL1, MDM2, MET, MMP1, MMP13, MMP2, MMP8, MMP9, NCOA1, NCOA2, NFKBIA, NOS2, NR3C1, NR3C2, NUF2, OPRD1, OPRM1, PCNA, PDE3A, PGR, PIK3CG, PIM1, PKIA, PKM, PLAU, PPARG, PPIF , PRSS1, PTGES, PTGS1, PTGS2, PTPN1, RB1, RELA, RXRA, SCN5A, SLC2A4, SLC6A2, SLC6A3, SLC6A4, TNF, TOP1, TOP2A, TP53, TUBB1, TYR, VEGFA, XDH
Radix Paeoniae Rubra	ADH1C, ADRA1A, ADRA1B, ADRA2A, ADRB1, ADRB2, AHR, AKT1, ALOX12, APOD, AR, ARG, BAX, BCL2, CASP3, CASP8, CASP9, CAT, CCNB1, CD14, CDKN1A, CHRM1, CHRM2, CHRM3, CHRM4, CHRNA2, CHRNA7, CTNNBIP1, CTRB1, CXCL8, CYCS,

	DHODH, DRD5, EGLN1, ESR1, F10, F2, FABP, FOS, FOSL1, FOSL2, GABRA1, GABRA3, GABRA5, GABRA6, GSTA1, GSTA2, GSTM1, GSTM2, GSTP1, HAS2, HIF1A, HTR2A, IGF2, IGHG1, IGHG2, IL6, JUN, KCNH2, LBP, LTA4H, MAOB, MAP2, MARS1, MMP2, MMP9, MPO, NCOA1, NCOA2, NFATC1, NFKBIA, NOS2, NOX5, NR3C2, OPRM1, PDE3A, PGR, PLAU, PON1, PPIF , PRKCA, PRKCB, PRSS1, PTER, PTGS1, PTGS2, PTPN1, RELA, RXRA, SCN5A, SLC6A2, SLC6A3, SLC6A4, TDRD7, TGFB1, TNF, TP53, VEGFA
Hedysarum Multijugum Maxim.	AARS1, ABAT, ABCA2, ACACA, ACHE, ACP3, ACTB, ADH1A, ADH1C, ADRA1A, ADRA1B, ADRA1D, ADRA2A, ADRA2B, ADRA2C, ADRB1, ADRB2, AHR, AHSA1, AKR1, AKR1C3, AKT1, ALB, ALOX5, APOB, AR, ARG, ATP5F1B, BAX, BCHE, BCL2, BCL2L1, BIRC5, CA2, CASP3, CASP8, CASP9, CAV1, CCL2, CCNA2, CCNB1, CCND1, CD40LG, CDC25B, CDKN1A, CHEK1, CHEK2, CHRM1, CHRM2, CHRM3, CHRM4, CHRM5, CHRNA7, CHUK, CLDN4, COL1A1, COL1A3, CRP, CTNNBIP1, CTRB1, CTSD, CXCL10, CXCL11, CXCL2, CXCL8, CYP1A1, CYP1A2, CYP1B1, CYP3A4, DAO, DCAF5, DHODH, DIO1, DRD5, DUOX2, E2F1, E2F2, EGF, EGFR, EIF6, ELK1, ERBB2, ERBB3, ESR1, ESR2, F10, F2, F3, F7, FOS, GABRA1, GABRA2, GABRA3, GABRA5, GABRA6, GARS1, GATM, GJA1, GOT1, GOT2, GRIA1, GRIA2, GRIK2, GRIN1, GRIN2B, GRP78, GSK3B, GSTM1, GSTM2, GSTP1, HAS2, HIF1A, HK2, HMOX1, HNF1A, HNF4A, HP, HSD3B1, HSD3B2, HSF1, HSPB1, HTR2A, HTR3A, ICAM1, IFNG, IGF2, IGFBP3, IGHG1, IGHG2, IKBKB, IL10, IL1A, IL1B, IL2, IL4, IL6, INSR, IRF1, JUN, KCNH2, KDR, LPCAT2, LPL, LTA4H, MAN2A1, MAOB, MAPK1, MAPK14, MAPK8, MARS1, ME2, MET, MGAM, MMP1, MMP2, MMP3, MMP9, MOGAT2, MPO, MT-ND6, MYC, NCF1, NCOA1, NCOA2, NFE2L2, NFKBIA, NKX3-1, NOS2, NOS3, NPEPPS, NQO1, NR1I2, NR1I3, ODC1, OLR1, OPRD1, OPRM1, P4HA1, PCNA, PCOLCE, PDE3A, PDHX, PGR, PIK3CG, PIM1, PKIA, PKM, PLAT, PLAU, PON1, POR, PPARA, PPARD, PPARD , PPARG, PPARGC1A, PPIA, PPIF , PPP3CA, PRKCA, PRKCB, PRSS1, PRSS3, PSMD3, PTEN, PTER, PTGER3, PTGS1, PTGS2, PTPN1, PYGM, RAF1, RASA1, RASSF1, RB1, RELA, REN, RUNX1T1, RUNX2, RXRA, RXRB, RXRG, SCN5A, SELE, SERPINE1, SIRT1, SLC2A4, SLC6A2, SLC6A3, SLC6A4, SLPI, SOD1, SPP1, STAT1, STE, TGFB1, THBD, TKFC , TNF, TNKS, TOP1, TOP-2, TOP2A, TP53, TPI1, TRIM26, TRPV1, TYRP1, UCP2, VCAM1, VEGFA, XDH

Table S3-1 KEGG Enrichment analysis of similar genes between target genes of KXS compound and essential genes of AD

Term	P Value
hsa05164:Influenza A	5.55×10^{-8}
hsa05140:Leishmaniasis	4.06×10^{-7}
hsa05133:Pertussis	5.36×10^{-7}
hsa04932:Non-alcoholic fatty liver disease (NAFLD)	6.77×10^{-7}
hsa05132:Salmonella infection	8.91×10^{-7}
hsa05010:Alzheimer's disease	1.27×10^{-6}
hsa05152:Tuberculosis	1.72×10^{-6}
hsa05142:Chagas disease (American trypanosomiasis)	2.74×10^{-6}
hsa04668:TNF signaling pathway	3.16×10^{-6}
hsa05145:Toxoplasmosis	3.62×10^{-6}
hsa04621:NOD-like receptor signaling pathway	6.67×10^{-6}
hsa04380:Osteoclast differentiation	8.56×10^{-6}
hsa05200:Pathways in cancer	1.37×10^{-5}
hsa05222:Small cell lung cancer	3.53×10^{-5}
hsa04024:cAMP signaling pathway	6.33×10^{-5}
hsa04620:Toll-like receptor signaling pathway	8.41×10^{-5}
hsa04014:Ras signaling pathway	1.19×10^{-4}
hsa04722:Neurotrophin signaling pathway	1.36×10^{-4}
hsa04010:MAPK signaling pathway	2.02×10^{-4}
hsa05160:Hepatitis C	2.03×10^{-4}
hsa05161:Hepatitis B	2.83×10^{-4}
hsa05210:Colorectal cancer	3.59×10^{-4}
hsa05131:Shigellosis	3.95×10^{-4}
hsa05212:Pancreatic cancer	4.13×10^{-4}
hsa04662:B cell receptor signaling pathway	4.93×10^{-4}
hsa04917:Prolactin signaling pathway	5.36×10^{-4}
hsa05168:Herpes simplex infection	6.86×10^{-4}
hsa04064:NF-kappa B signaling pathway	9.71×10^{-4}
hsa05215:Prostate cancer	0.001004329
hsa04660:T cell receptor signaling pathway	0.001454886
hsa04931:Insulin resistance	0.001816109
hsa04725:Cholinergic synapse	0.0019648
hsa05020:Prion diseases	0.002724775
hsa05162:Measles	0.003290683
hsa04068:FoxO signaling pathway	0.003361258
hsa04910:Insulin signaling pathway	0.003652913
hsa04930:Type II diabetes mellitus	0.005375755
hsa05030:Cocaine addiction	0.005596926
hsa05014:Amyotrophic lateral sclerosis (ALS)	0.005822243
hsa05134:Legionellosis	0.006764598
hsa04151:PI3K-Akt signaling pathway	0.006998406
hsa05221:Acute myeloid leukemia	0.007260181

hsa04020:Calcium signaling pathway	0.007558119
hsa04062:Chemokine signaling pathway	0.008401243
hsa04210:Apoptosis	0.008842847
hsa04623:Cytosolic DNA-sensing pathway	0.00940191
hsa05321:Inflammatory bowel disease (IBD)	0.00940191
hsa05031:Amphetamine addiction	0.009976494
hsa04720:Long-term potentiation	0.009976494
hsa05120:Epithelial cell signaling in Helicobacter pylori infection	0.010269564
hsa04920:Adipocytokine signaling pathway	0.01117168
hsa04622:RIG-I-like receptor signaling pathway	0.01117168
hsa05220:Chronic myeloid leukemia	0.011792009
hsa04012:ErbB signaling pathway	0.016912655
hsa04713:Circadian entrainment	0.019968916
hsa04066:HIF-1 signaling pathway	0.020366278
hsa04723:Retrograde endocannabinoid signaling	0.022403087
hsa04080:Neuroactive ligand-receptor interaction	0.024507326
hsa05146:Amoebiasis	0.024521802
hsa04726:Serotonergic synapse	0.026720505
hsa04724:Glutamatergic synapse	0.028077335
hsa04071:Sphingolipid signaling pathway	0.030873734
hsa05169:Epstein-Barr virus infection	0.031829949
hsa04728:Dopaminergic synapse	0.034769215

Table S3-2 GO Enrichment analysis (BP) of similar genes between target genes of KXS compound and essential genes of AD

Term	P Value
GO:0006954~inflammatory response	5.87×10^{-4}
GO:0051092~positive regulation of NF-kappaB transcription factor activity	0.00169
GO:0003056~regulation of vascular smooth muscle contraction	0.001904
GO:0031622~positive regulation of fever generation	0.00238
GO:0046541~saliva secretion	0.00238
GO:1903140~regulation of establishment of endothelial barrier	0.003331
GO:0007197~adenylate cyclase-inhibiting G-protein coupled acetylcholine receptor signaling pathway	0.003331
GO:0007207~phospholipase C-activating G-protein coupled acetylcholine receptor signaling pathway	0.003806
GO:0035729~cellular response to hepatocyte growth factor stimulus	0.006652
GO:0007213~G-protein coupled acetylcholine receptor signaling pathway	0.007125
GO:0007399~nervous system development	0.007615
GO:0045944~positive regulation of transcription from RNA polymerase II promoter	0.008917
GO:0046827~positive regulation of protein export from nucleus	0.009018

GO:0042346~positive regulation of NF-kappaB import into nucleus	0.009963
GO:0045987~positive regulation of smooth muscle contraction	0.009963
GO:0008283~cell proliferation	0.012162
GO:0010575~positive regulation of vascular endothelial growth factor production	0.012794
GO:0046627~negative regulation of insulin receptor signaling pathway	0.013736
GO:0042177~negative regulation of protein catabolic process	0.015147
GO:0097192~extrinsic apoptotic signaling pathway in absence of ligand	0.016087
GO:0007271~synaptic transmission, cholinergic	0.017496
GO:0043066~negative regulation of apoptotic process	0.018409
GO:0007566~embryo implantation	0.019839
GO:0045429~positive regulation of nitric oxide biosynthetic process	0.020307
GO:0045893~positive regulation of transcription, DNA-templated	0.023253
GO:0050727~regulation of inflammatory response	0.029629
GO:0050796~regulation of insulin secretion	0.031484
GO:0035690~cellular response to drug	0.032411
GO:0071260~cellular response to mechanical stimulus	0.033336
GO:0007623~circadian rhythm	0.035185
GO:0009887~organ morphogenesis	0.043008
GO:0002223~stimulatory C-type lectin receptor signaling pathway	0.048953
GO:0006464~cellular protein modification process	0.048953

Table S3-3 GO Enrichment analysis (CC) of similar genes between target genes of KXS compound and essential genes of AD

Term	P Value
GO:0005829~cytosol	0.001484
GO:0048471~perinuclear region of cytoplasm	0.015285
GO:0005776~autophagosome	0.024977
GO:0045211~postsynaptic membrane	0.03854
GO:0031410~cytoplasmic vesicle	0.04749

Table S3-4 GO Enrichment analysis (MF) of similar genes between target genes of KXS compound and essential genes of AD

Term	P Value
GO:0016907~G-protein coupled acetylcholine receptor activity	0.005574
GO:0005149~interleukin-1 receptor binding	0.007161

Table S4-1 KEGG Enrichment analysis of similar genes between target genes of DGSYS compound and essential genes of AD

Term	P Value

hsa04024:cAMP signaling pathway	7.65×10^{-7}
hsa05142:Chagas disease (American trypanosomiasis)	8.38×10^{-7}
hsa04668:TNF signaling pathway	9.66×10^{-7}
hsa04014:Ras signaling pathway	1.67×10^{-6}
hsa04621:NOD-like receptor signaling pathway	2.67×10^{-6}
hsa05140:Leishmaniasis	6.95×10^{-6}
hsa05133:Pertussis	8.65×10^{-6}
hsa05010:Alzheimer's disease	8.99×10^{-6}
hsa05164:Influenza A	1.07×10^{-5}
hsa05132:Salmonella infection	1.3×10^{-5}
hsa04620:Toll-like receptor signaling pathway	3.42×10^{-5}
hsa05145:Toxoplasmosis	3.96×10^{-5}
hsa05200:Pathways in cancer	4.07×10^{-5}
hsa04722:Neurotrophin signaling pathway	5.58×10^{-5}
hsa04380:Osteoclast differentiation	7.86×10^{-5}
hsa05160:Hepatitis C	8.34×10^{-5}
hsa05030:Cocaine addiction	9.24×10^{-5}
hsa04932:Non-alcoholic fatty liver disease (NAFLD)	0.000137
hsa05131:Shigellosis	0.000206
hsa05212:Pancreatic cancer	0.000215
hsa04720:Long-term potentiation	0.000225
hsa05152:Tuberculosis	0.000253
hsa04662:B cell receptor signaling pathway	0.000257
hsa04020:Calcium signaling pathway	0.000264
hsa04917:Prolactin signaling pathway	0.00028
hsa05222:Small cell lung cancer	0.000476
hsa04064:NF-kappa B signaling pathway	0.00051
hsa05215:Prostate cancer	0.000527
hsa04713:Circadian entrainment	0.00066
hsa04660:T cell receptor signaling pathway	0.000767
hsa04931:Insulin resistance	0.00096
hsa04010:MAPK signaling pathway	0.000982
hsa04725:Cholinergic synapse	0.001039
hsa04724:Glutamatergic synapse	0.001123
hsa04080:Neuroactive ligand-receptor interaction	0.001379
hsa04728:Dopaminergic synapse	0.00157
hsa04910:Insulin signaling pathway	0.00195
hsa05161:Hepatitis B	0.002247
hsa05033:Nicotine addiction	0.00247
hsa04151:PI3K-Akt signaling pathway	0.003098
hsa04930:Type II diabetes mellitus	0.003541
hsa05014:Amyotrophic lateral sclerosis (ALS)	0.003837
hsa05034:Alcoholism	0.00396

hsa05168:Herpes simplex infection	0.004351
hsa04062:Chemokine signaling pathway	0.004554
hsa05221:Acute myeloid leukemia	0.004794
hsa05210:Colorectal cancer	0.005849
hsa04623:Cytosolic DNA-sensing pathway	0.006222
hsa04015:Rap1 signaling pathway	0.006396
hsa05031:Amphetamine addiction	0.006606
hsa05120:Epithelial cell signaling in Helicobacter pylori infection	0.006802
hsa04920:Adipocytokine signaling pathway	0.007406
hsa04622:RIG-I-like receptor signaling pathway	0.007406
hsa05220:Chronic myeloid leukemia	0.007822
hsa04012:ErbB signaling pathway	0.011266
hsa04066:HIF-1 signaling pathway	0.013602
hsa04723:Retrograde endocannabinoid signaling	0.014984
hsa05146:Amoebiasis	0.016424
hsa04071:Sphingolipid signaling pathway	0.020761
hsa05169:Epstein-Barr virus infection	0.021416
hsa05162:Measles	0.025173
hsa04068:FoxO signaling pathway	0.025528

Table S4-2 GO Enrichment analysis (BP) of similar genes between target genes of DGSYS compound and essential genes of AD

Term	P Value
GO:0019233~sensory perception of pain	7.84×10^{-6}
GO:0000165~MAPK cascade	3.71×10^{-5}
GO:0035235~ionotropic glutamate receptor signaling pathway	0.000151
GO:0042177~negative regulation of protein catabolic process	0.000271
GO:0038095~Fc-epsilon receptor signaling pathway	0.00031
GO:0007268~chemical synaptic transmission	0.000742
GO:0006954~inflammatory response	0.002757
GO:0045471~response to ethanol	0.002888
GO:0000187~activation of MAPK activity	0.002997
GO:0003056~regulation of vascular smooth muscle contraction	0.003093
GO:0046541~saliva secretion	0.003865
GO:0031622~positive regulation of fever generation	0.003865
GO:0051092~positive regulation of NF-kappaB transcription factor activity	0.004587
GO:0006468~protein phosphorylation	0.004644
GO:0007197~adenylate cyclase-inhibiting G-protein coupled acetylcholine receptor signaling pathway	0.005408
GO:1903140~regulation of establishment of endothelial barrier	0.005408
GO:0045944~positive regulation of transcription from RNA polymerase II promoter	0.005411

GO:0050852~T cell receptor signaling pathway	0.005647
GO:0007207~phospholipase C-activating G-protein coupled acetylcholine receptor signaling pathway	0.006178
GO:0045893~positive regulation of transcription, DNA-templated	0.00652
GO:0043547~positive regulation of GTPase activity	0.008422
GO:0007165~signal transduction	0.009811
GO:0035729~cellular response to hepatocyte growth factor stimulus	0.010788
GO:0007215~glutamate receptor signaling pathway	0.011555
GO:0007213~G-protein coupled acetylcholine receptor signaling pathway	0.011555
GO:0046827~positive regulation of protein export from nucleus	0.014615
GO:0045987~positive regulation of smooth muscle contraction	0.016142
GO:0042346~positive regulation of NF-kappaB import into nucleus	0.016142
GO:0043278~response to morphine	0.017667
GO:0051090~regulation of sequence-specific DNA binding transcription factor activity	0.019189
GO:0007399~nervous system development	0.020053
GO:0010575~positive regulation of vascular endothelial growth factor production	0.02071
GO:0010800~positive regulation of peptidyl-threonine phosphorylation	0.02071
GO:0060079~excitatory postsynaptic potential	0.021469
GO:0043065~positive regulation of apoptotic process	0.02179
GO:0046627~negative regulation of insulin receptor signaling pathway	0.022228
GO:0042493~response to drug	0.022337
GO:0001975~response to amphetamine	0.023744
GO:0031663~lipopolysaccharide-mediated signaling pathway	0.024501
GO:0097192~extrinsic apoptotic signaling pathway in absence of ligand	0.026014
GO:0007611~learning or memory	0.027524
GO:0007271~synaptic transmission, cholinergic	0.028279
GO:0018107~peptidyl-threonine phosphorylation	0.029033
GO:0060291~long-term synaptic potentiation	0.029033
GO:0008283~cell proliferation	0.031534
GO:0007566~embryo implantation	0.032043
GO:0045429~positive regulation of nitric oxide biosynthetic process	0.032794
GO:0008542~visual learning	0.034295
GO:0043066~negative regulation of apoptotic process	0.04691
GO:0007613~memory	0.046966
GO:0050727~regulation of inflammatory response	0.047707
GO:0009611~response to wounding	0.047707
GO:0008284~positive regulation of cell proliferation	0.048974

Table S4-3 GO Enrichment analysis (CC) of similar genes between target genes of DGSYS compound and essential genes of AD

Term	P Value
GO:0030054~cell junction	1.08×10^{-5}
GO:0045211~postsynaptic membrane	1.15×10^{-5}
GO:0017146~NMDA selective glutamate receptor complex	2.57×10^{-5}
GO:0045202~synapse	0.000256
GO:0014069~postsynaptic density	0.000269
GO:0043005~neuron projection	0.000564
GO:0032279~asymmetric synapse	0.005694
GO:0043083~synaptic cleft	0.005694
GO:0009986~cell surface	0.005989
GO:0005622~intracellular	0.011902
GO:0005886~plasma membrane	0.014002
GO:0005887~integral component of plasma membrane	0.014654
GO:0005829~cytosol	0.01924
GO:0030425~dendrite	0.022988
GO:0043679~axon terminus	0.033716
GO:0005901~caveola	0.045403

Table S4-4 GO Enrichment analysis (MF) of similar genes between target genes of DGSYS compound and essential genes of AD

Term	P Value
GO:0004972~NMDA glutamate receptor activity	1.53×10^{-5}
GO:0005234~extracellular-glutamate-gated ion channel activity	8.32×10^{-5}
GO:0042803~protein homodimerization activity	0.001814
GO:0005088~Ras guanyl-nucleotide exchange factor activity	0.003417
GO:0016907~G-protein coupled acetylcholine receptor activity	0.005379
GO:0004707~MAP kinase activity	0.010732
GO:0016594~glycine binding	0.011494
GO:0004970~ionotropic glutamate receptor activity	0.011494
GO:0005515~protein binding	0.016236
GO:0042166~acetylcholine binding	0.019846
GO:0004435~phosphatidylinositol phospholipase C activity	0.020601
GO:0051059~NF-kappaB binding	0.022866
GO:0004672~protein kinase activity	0.030134
GO:0004674~protein serine/threonine kinase activity	0.032818
GO:0019901~protein kinase binding	0.032818

Table S5-1 KEGG Enrichment analysis of similar genes between target genes of YGS compound and essential genes of AD

Term	P Value
hsa05140:Leishmaniasis	2.81×10^{-7}

hsa05133:Pertussis	3.71×10^{-7}
hsa05132:Salmonella infection	6.19×10^{-7}
hsa05010:Alzheimer's disease	8.09×10^{-7}
hsa05164:Influenza A	9.95×10^{-7}
hsa05142:Chagas disease (American trypanosomiasis)	1.91×10^{-6}
hsa04024:cAMP signaling pathway	2.13×10^{-6}
hsa04668:TNF signaling pathway	2.2×10^{-6}
hsa04014:Ras signaling pathway	4.59×10^{-6}
hsa04621:NOD-like receptor signaling pathway	5.03×10^{-6}
hsa04380:Osteoclast differentiation	5.98×10^{-6}
hsa04932:Non-alcoholic fatty liver disease (NAFLD)	1.2×10^{-5}
hsa05152:Tuberculosis	2.6×10^{-5}
hsa04620:Toll-like receptor signaling pathway	6.38×10^{-5}
hsa05145:Toxoplasmosis	7.38×10^{-5}
hsa04722:Neurotrophin signaling pathway	0.000104
hsa05200:Pathways in cancer	0.000108
hsa04010:MAPK signaling pathway	0.000143
hsa05030:Cocaine addiction	0.000146
hsa05160:Hepatitis C	0.000155
hsa05131:Shigellosis	0.000323
hsa05212:Pancreatic cancer	0.000338
hsa04720:Long-term potentiation	0.000354
hsa04662:B cell receptor signaling pathway	0.000403
hsa04917:Prolactin signaling pathway	0.000439
hsa04020:Calcium signaling pathway	0.000483
hsa05222:Small cell lung cancer	0.000744
hsa04064:NF-kappa B signaling pathway	0.000797
hsa05215:Prostate cancer	0.000824
hsa04713:Circadian entrainment	0.001029
hsa04660:T cell receptor signaling pathway	0.001195
hsa04931:Insulin resistance	0.001493
hsa04725:Cholinergic synapse	0.001615
hsa04724:Glutamatergic synapse	0.001744
hsa05020:Prion diseases	0.002392
hsa04728:Dopaminergic synapse	0.002431
hsa04080:Neuroactive ligand-receptor interaction	0.00247
hsa05162:Measles	0.002712
hsa04910:Insulin signaling pathway	0.003012
hsa05033:Nicotine addiction	0.0033
hsa05161:Hepatitis B	0.003466
hsa04930:Type II diabetes mellitus	0.004725
hsa05014:Amyotrophic lateral sclerosis (ALS)	0.005118
hsa04151:PI3K-Akt signaling pathway	0.005462

hsa05034:Alcoholism	0.006067
hsa05221:Acute myeloid leukemia	0.006386
hsa05168:Herpes simplex infection	0.006656
hsa04062:Chemokine signaling pathway	0.006963
hsa05210:Colorectal cancer	0.007782
hsa05321:Inflammatory bowel disease (IBD)	0.008276
hsa04623:Cytosolic DNA-sensing pathway	0.008276
hsa05031:Amphetamine addiction	0.008783
hsa05120:Epithelial cell signaling in Helicobacter pylori infection	0.009042
hsa04015:Rap1 signaling pathway	0.009727
hsa04920:Adipocytokine signaling pathway	0.009839
hsa04622:RIG-I-like receptor signaling pathway	0.009839
hsa05220:Chronic myeloid leukemia	0.010388
hsa04012:ErbB signaling pathway	0.01492
hsa04066:HIF-1 signaling pathway	0.017982
hsa04723:Retrograde endocannabinoid signaling	0.01979
hsa05146:Amoebiasis	0.021672
hsa04071:Sphingolipid signaling pathway	0.027322
hsa05169:Epstein-Barr virus infection	0.028173
hsa04068:FoxO signaling pathway	0.033506

Table S5-2 GO Enrichment analysis (BP) of similar genes between target genes of YGS compound and essential genes of AD

Term	P Value
GO:0019233~sensory perception of pain	1.24×10^{-5}
GO:0000165~MAPK cascade	6.9×10^{-5}
GO:0035235~ionotropic glutamate receptor signaling pathway	0.000203
GO:0010575~positive regulation of vascular endothelial growth factor production	0.000258
GO:0006954~inflammatory response	0.000286
GO:0042177~negative regulation of protein catabolic process	0.000364
GO:0097192~extrinsic apoptotic signaling pathway in absence of ligand	0.000411
GO:0038095~Fc-epsilon receptor signaling pathway	0.000485
GO:0007268~chemical synaptic transmission	0.001155
GO:0045944~positive regulation of transcription from RNA polymerase II promoter	0.001236
GO:0001660~fever generation	0.002678
GO:0003056~regulation of vascular smooth muscle contraction	0.003569
GO:0008283~cell proliferation	0.003846
GO:0045471~response to ethanol	0.003856
GO:0000187~activation of MAPK activity	0.004001
GO:0031622~positive regulation of fever generation	0.004459

GO:0046541~saliva secretion	0.004459
GO:0019221~cytokine-mediated signaling pathway	0.005934
GO:0051092~positive regulation of NF-kappaB transcription factor activity	0.006111
GO:1903140~regulation of establishment of endothelial barrier	0.006237
GO:0071639~positive regulation of monocyte chemotactic protein-1 production	0.006237
GO:0007197~adenylate cyclase-inhibiting G-protein coupled acetylcholine receptor signaling pathway	0.006237
GO:0006468~protein phosphorylation	0.007097
GO:0007207~phospholipase C-activating G-protein coupled acetylcholine receptor signaling pathway	0.007125
GO:0035234~ectopic germ cell programmed cell death	0.007125
GO:0050852~T cell receptor signaling pathway	0.007515
GO:0043123~positive regulation of I-kappaB kinase/NF-kappaB signaling	0.008838
GO:0045893~positive regulation of transcription, DNA-templated	0.009911
GO:0045086~positive regulation of interleukin-2 biosynthetic process	0.01067
GO:0070498~interleukin-1-mediated signaling pathway	0.012438
GO:0035729~cellular response to hepatocyte growth factor stimulus	0.012438
GO:0043547~positive regulation of GTPase activity	0.012746
GO:0007215~glutamate receptor signaling pathway	0.013321
GO:0007213~G-protein coupled acetylcholine receptor signaling pathway	0.013321
GO:0007165~signal transduction	0.016768
GO:0046827~positive regulation of protein export from nucleus	0.016846
GO:0045987~positive regulation of smooth muscle contraction	0.018603
GO:0042346~positive regulation of NF-kappaB import into nucleus	0.018603
GO:0043278~response to morphine	0.020358
GO:0051090~regulation of sequence-specific DNA binding transcription factor activity	0.02211
GO:0045840~positive regulation of mitotic nuclear division	0.022985
GO:0010800~positive regulation of peptidyl-threonine phosphorylation	0.023859
GO:0060079~excitatory postsynaptic potential	0.024732
GO:0046627~negative regulation of insulin receptor signaling pathway	0.025605
GO:0007399~nervous system development	0.026397
GO:0001975~response to amphetamine	0.027348
GO:0031663~lipopolysaccharide-mediated signaling pathway	0.028218
GO:0043065~positive regulation of apoptotic process	0.028655
GO:0042493~response to drug	0.029365
GO:0007611~learning or memory	0.031693
GO:0050714~positive regulation of protein secretion	0.031693
GO:2001240~negative regulation of extrinsic apoptotic signaling pathway in absence of ligand	0.03256
GO:0007271~synaptic transmission, cholinergic	0.03256
GO:0018107~peptidyl-threonine phosphorylation	0.033426
GO:0060291~long-term synaptic potentiation	0.033426

GO:0007566~embryo implantation	0.036883
GO:0045429~positive regulation of nitric oxide biosynthetic process	0.037746
GO:0032755~positive regulation of interleukin-6 production	0.039468
GO:0008542~visual learning	0.039468
GO:0051781~positive regulation of cell division	0.041188
GO:0008285~negative regulation of cell proliferation	0.047571

Table S5-3 GO Enrichment analysis (CC) of similar genes between target genes of YGS compound and essential genes of AD

Term	P Value
GO:0045211~postsynaptic membrane	2.16×10^{-5}
GO:0030054~cell junction	2.42×10^{-5}
GO:0017146~NMDA selective glutamate receptor complex	3.46×10^{-5}
GO:0045202~synapse	0.000402
GO:0014069~postsynaptic density	0.000421
GO:0009986~cell surface	0.000813
GO:0043005~neuron projection	0.00088
GO:0005887~integral component of plasma membrane	0.00435
GO:0032279~asymmetric synapse	0.006567
GO:0043083~synaptic cleft	0.006567
GO:0005886~plasma membrane	0.009282
GO:0005829~cytosol	0.010745
GO:0005622~intracellular	0.020214
GO:0030425~dendrite	0.03021
GO:0043679~axon terminus	0.038803

Table S5-4 GO Enrichment analysis (MF) of similar genes between target genes of YGS compound and essential genes of AD

Term	P Value
GO:0004972~NMDA glutamate receptor activity	2.06×10^{-5}
GO:0005234~extracellular-glutamate-gated ion channel activity	0.000112
GO:0042803~protein homodimerization activity	0.003231
GO:0005088~Ras guanyl-nucleotide exchange factor activity	0.004559
GO:0005515~protein binding	0.005766
GO:0016907~G-protein coupled acetylcholine receptor activity	0.006205
GO:0005149~interleukin-1 receptor binding	0.011494
GO:0004707~MAP kinase activity	0.012373
GO:0016594~glycine binding	0.013251
GO:0004970~ionotropic glutamate receptor activity	0.013251
GO:0042166~acetylcholine binding	0.022865
GO:0004435~phosphatidylinositol phospholipase C activity	0.023734

GO:0051059~NF-kappaB binding	0.026339
GO:0001540~beta-amyloid binding	0.029801
GO:0004672~protein kinase activity	0.039453
GO:0004674~protein serine/threonine kinase activity	0.04291
GO:0019901~protein kinase binding	0.04291

Table S6-1 KEGG Enrichment analysis of similar genes between target genes of YQTYT compound and essential genes of AD

Term	P Value
hsa05164:Influenza A	5.55×10^{-8}
hsa05140:Leishmaniasis	4.06×10^{-7}
hsa05133:Pertussis	5.36×10^{-7}
hsa04932:Non-alcoholic fatty liver disease (NAFLD)	6.77×10^{-7}
hsa05132:Salmonella infection	8.91×10^{-7}
hsa05010:Alzheimer's disease	1.27×10^{-6}
hsa05152:Tuberculosis	1.72×10^{-6}
hsa05142:Chagas disease (American trypanosomiasis)	2.74×10^{-6}
hsa04668:TNF signaling pathway	3.16×10^{-6}
hsa05145:Toxoplasmosis	3.62×10^{-6}
hsa04621:NOD-like receptor signaling pathway	6.67×10^{-6}
hsa04380:Osteoclast differentiation	8.56×10^{-6}
hsa05200:Pathways in cancer	1.37×10^{-5}
hsa05222:Small cell lung cancer	3.53×10^{-5}
hsa04024:cAMP signaling pathway	6.33×10^{-5}
hsa04620:Toll-like receptor signaling pathway	8.41×10^{-5}
hsa04014:Ras signaling pathway	0.000119
hsa04722:Neurotrophin signaling pathway	0.000136
hsa04010:MAPK signaling pathway	0.000202
hsa05160:Hepatitis C	0.000203
hsa05161:Hepatitis B	0.000283
hsa05210:Colorectal cancer	0.000359
hsa05131:Shigellosis	0.000395
hsa05212:Pancreatic cancer	0.000413
hsa04662:B cell receptor signaling pathway	0.000493
hsa04917:Prolactin signaling pathway	0.000536
hsa05168:Herpes simplex infection	0.000686
hsa04064:NF-kappa B signaling pathway	0.000971
hsa05215:Prostate cancer	0.001004
hsa04660:T cell receptor signaling pathway	0.001455
hsa04931:Insulin resistance	0.001816
hsa04725:Cholinergic synapse	0.001965
hsa05020:Prion diseases	0.002725

hsa05162:Measles	0.003291
hsa04068:FoxO signaling pathway	0.003361
hsa04910:Insulin signaling pathway	0.003653
hsa04930:Type II diabetes mellitus	0.005376
hsa05030:Cocaine addiction	0.005597
hsa05014:Amyotrophic lateral sclerosis (ALS)	0.005822
hsa05134:Legionellosis	0.006765
hsa04151:PI3K-Akt signaling pathway	0.006998
hsa05221:Acute myeloid leukemia	0.00726
hsa04020:Calcium signaling pathway	0.007558
hsa04062:Chemokine signaling pathway	0.008401
hsa04210:Apoptosis	0.008843
hsa04623:Cytosolic DNA-sensing pathway	0.009402
hsa05321:Inflammatory bowel disease (IBD)	0.009402
hsa05031:Amphetamine addiction	0.009976
hsa04720:Long-term potentiation	0.009976
hsa05120:Epithelial cell signaling in Helicobacter pylori infection	0.01027
hsa04920:Adipocytokine signaling pathway	0.011172
hsa04622:RIG-I-like receptor signaling pathway	0.011172
hsa05220:Chronic myeloid leukemia	0.011792
hsa04012:ErbB signaling pathway	0.016913
hsa04713:Circadian entrainment	0.019969
hsa04066:HIF-1 signaling pathway	0.020366
hsa04723:Retrograde endocannabinoid signaling	0.022403
hsa04080:Neuroactive ligand-receptor interaction	0.024507
hsa05146:Amoebiasis	0.024522
hsa04726:Serotonergic synapse	0.026721
hsa04724:Glutamatergic synapse	0.028077
hsa04071:Sphingolipid signaling pathway	0.030874
hsa05169:Epstein-Barr virus infection	0.03183
hsa04728:Dopaminergic synapse	0.034769

Table S6-2 GO Enrichment analysis (BP) of similar genes between target genes of YQTYT compound and essential genes of AD

Term	P Value
GO:0045944~positive regulation of transcription from RNA polymerase II promoter	1.63×10^{-5}
GO:0035235~ionotropic glutamate receptor signaling pathway	0.000232
GO:0010575~positive regulation of vascular endothelial growth factor production	0.000295
GO:0006954~inflammatory response	0.000375
GO:0097192~extrinsic apoptotic signaling pathway in absence of ligand	0.000469

GO:0038095~Fc-epsilon receptor signaling pathway	0.000592
GO:0006468~protein phosphorylation	0.000753
GO:0019233~sensory perception of pain	0.001098
GO:0000165~MAPK cascade	0.001809
GO:1900034~regulation of cellular response to heat	0.002268
GO:0007399~nervous system development	0.002346
GO:0001660~fever generation	0.002856
GO:0003056~regulation of vascular smooth muscle contraction	0.003806
GO:0000187~activation of MAPK activity	0.004554
GO:0008283~cell proliferation	0.004657
GO:0046541~saliva secretion	0.004756
GO:0031622~positive regulation of fever generation	0.004756
GO:0006979~response to oxidative stress	0.004806
GO:0071356~cellular response to tumor necrosis factor	0.004806
GO:0045766~positive regulation of angiogenesis	0.00524
GO:0071639~positive regulation of monocyte chemotactic protein-1 production	0.006652
GO:0007197~adenylate cyclase-inhibiting G-protein coupled acetylcholine receptor signaling pathway	0.006652
GO:1903140~regulation of establishment of endothelial barrier	0.006652
GO:0019221~cytokine-mediated signaling pathway	0.006747
GO:0051092~positive regulation of NF-kappaB transcription factor activity	0.006948
GO:0007207~phospholipase C-activating G-protein coupled acetylcholine receptor signaling pathway	0.007599
GO:0035234~ectopic germ cell programmed cell death	0.007599
GO:0043066~negative regulation of apoptotic process	0.008509
GO:0050852~T cell receptor signaling pathway	0.008539
GO:0008284~positive regulation of cell proliferation	0.009085
GO:0043123~positive regulation of I-kappaB kinase/NF-kappaB signaling	0.010038
GO:0045086~positive regulation of interleukin-2 biosynthetic process	0.011378
GO:0045893~positive regulation of transcription, DNA-templated	0.011924
GO:0001967~suckling behavior	0.012321
GO:0070498~interleukin-1-mediated signaling pathway	0.013262
GO:0035729~cellular response to hepatocyte growth factor stimulus	0.013262
GO:0007213~G-protein coupled acetylcholine receptor signaling pathway	0.014203
GO:0046827~positive regulation of protein export from nucleus	0.017959
GO:0042346~positive regulation of NF-kappaB import into nucleus	0.019832
GO:0045987~positive regulation of smooth muscle contraction	0.019832
GO:0007165~signal transduction	0.021159
GO:0007268~chemical synaptic transmission	0.021395
GO:0043278~response to morphine	0.021701
GO:0051090~regulation of sequence-specific DNA binding transcription factor activity	0.023567
GO:0045840~positive regulation of mitotic nuclear division	0.024499

GO:0010800~positive regulation of peptidyl-threonine phosphorylation	0.02543
GO:0046627~negative regulation of insulin receptor signaling pathway	0.027289
GO:0031663~lipopolysaccharide-mediated signaling pathway	0.030072
GO:0042177~negative regulation of protein catabolic process	0.030072
GO:0043065~positive regulation of apoptotic process	0.032368
GO:0050714~positive regulation of protein secretion	0.033771
GO:0007271~synaptic transmission, cholinergic	0.034693
GO:2001240~negative regulation of extrinsic apoptotic signaling pathway in absence of ligand	0.034693
GO:0018107~peptidyl-threonine phosphorylation	0.035615
GO:0007566~embryo implantation	0.039294
GO:0045429~positive regulation of nitric oxide biosynthetic process	0.040212
GO:0032755~positive regulation of interleukin-6 production	0.042045
GO:0008542~visual learning	0.042045
GO:0051781~positive regulation of cell division	0.043874
GO:0008344~adult locomotory behavior	0.048434

Table S6-3 GO Enrichment analysis (CC) of similar genes between target genes of YQTYT compound and essential genes of AD

Term	P Value
GO:0045202~synapse	1.56×10^{-5}
GO:0030054~cell junction	0.000568
GO:0005829~cytosol	0.000715
GO:0045211~postsynaptic membrane	0.000767
GO:0043083~synaptic cleft	0.007004
GO:0032279~asymmetric synapse	0.007004
GO:0017146~NMDA selective glutamate receptor complex	0.009618
GO:0009986~cell surface	0.010975
GO:0014069~postsynaptic density	0.011086
GO:0048471~perinuclear region of cytoplasm	0.015833
GO:0043005~neuron projection	0.017924
GO:0005739~mitochondrion	0.025364
GO:0005622~intracellular	0.025427
GO:0043198~dendritic shaft	0.027739
GO:0005887~integral component of plasma membrane	0.030966
GO:0030425~dendrite	0.034113
GO:0043679~axon terminus	0.041337
GO:0031594~neuromuscular junction	0.047229
GO:0005886~plasma membrane	0.049323

Table S6-4 GO Enrichment analysis (MF) of similar genes between target genes of YQTYT

compound and essential genes of AD

Term	P Value
GO:0005515~protein binding	0.003393
GO:0042803~protein homodimerization activity	0.004162
GO:0016907~G-protein coupled acetylcholine receptor activity	0.006617
GO:0020037~heme binding	0.007283
GO:0004972~NMDA glutamate receptor activity	0.007559
GO:0005149~interleukin-1 receptor binding	0.012256
GO:0004707~MAP kinase activity	0.013193
GO:0016594~glycine binding	0.014129
GO:0005234~extracellular-glutamate-gated ion channel activity	0.016932
GO:0042166~acetylcholine binding	0.024371
GO:0004435~phosphatidylinositol phospholipase C activity	0.025297
GO:0051059~NF-kappaB binding	0.028071
GO:0008134~transcription factor binding	0.028975
GO:0042802~identical protein binding	0.031631
GO:0019899~enzyme binding	0.0388
GO:0005102~receptor binding	0.043133
GO:0004672~protein kinase activity	0.044468
GO:0019901~protein kinase binding	0.048333
GO:0004674~protein serine/threonine kinase activity	0.048333
